# Characterization of the promoter region of the bovine long-chain acyl-CoA synthetase 1 gene: Roles of E2F1, Sp1, KLF15, and E2F4

**DOI:** 10.1038/srep19661

**Published:** 2016-01-19

**Authors:** Zhi-Dong Zhao, Lin-Sen Zan, An-Ning Li, Gong Cheng, Shi-Jun Li, Ya-Ran Zhang, Xiao-Yu Wang, Ying-Ying Zhang

**Affiliations:** 1 College of Animal Science and Technology, Northwest A&F University, Yangling 712100 Shaanxi, People’s Republic of China; 2 National Beef Cattle Improvement Center, Northwest A&F University, Yangling 712100 Shaanxi, People’s Republic of China

## Abstract

The nutritional value and eating qualities of beef are enhanced when the unsaturated fatty acid content of fat is increased. Long-chain acyl-CoA synthetase 1 (ACSL1) plays key roles in fatty acid transport and degradation, as well as lipid synthesis. It has been identified as a plausible functional and positional candidate gene for manipulations of fatty acid composition in bovine skeletal muscle. In the present study, we determined that bovine ACSL1was highly expressed in subcutaneous adipose tissue and *longissimus thoracis*. To elucidate the molecular mechanisms involved in bovine ACSL1 regulation, we cloned and characterized the promoter region of ACSL1. Applying 5′-rapid amplification of cDNA end analysis (RACE), we identified multiple transcriptional start sites (TSSs) in its promoter region. Using a series of 5′ deletion promoter plasmids in luciferase reporter assays, we found that the proximal minimal promoter of ACSL1 was located within the region −325/−141 relative to the TSS and it was also located in the predicted CpG island. Mutational analysis and electrophoretic mobility shift assays demonstrated that E2F1, Sp1, KLF15 and E2F4 binding to the promoter region drives ACSL1 transcription. Together these interactions integrate and frame a key functional role for ACSL1 in mediating the lipid composition of beef.

The nutritional value and eating qualities of beef are enhanced when the unsaturated fatty acid content of fat is increased. Longer chain (n-3) polyunsaturated fatty acids such as docosahexaenoic acid (DHA; 22:6, n-3) and eicosapentaenoic acid (EPA, 20:5, n-3) also have well-recognized beneficial effects in terms of reducing the risk of cardiovascular disease, cancer, and type-2 diabetes, as well as critical roles in brain function, visual development in the fetus, and the lifelong maintenance of neural and visual tissues^1^,^2^,^3^,^4^. Long-chain acyl-CoA synthetase 1 (ACSL1) has been identified as a plausible functional and positional candidate gene for manipulation of fatty acid composition in bovine skeletal muscle. Polymorphisms of ACSL1 gene have the most significant associations with the relative contents of distinct fractions and the ratios of fatty acids (e.g., n-3 fatty acids, polyunsaturated, (n-3) long-chain polyunsaturated fatty acids, and trans-vaccenic acid) in bovine skeletal muscle^5^.

Intramuscular fat deposition and the fatty acid profiles of beef are determined mainly by lipid metabolism, which dictates the balance between fat deposition and fat oxidation in skeletal muscle. ACSL1 belongs to the class of acyl-CoA synthetases, which are essential for the activation, transport, and degradation of fatty acids, as well as lipid synthesis^6^. ACSL1 is also known to be localized to glucose transporter 4-(GLUT4) containing vesicles^7^. ACSL1 deficiency impairs fasting glucose homeostasis in muscle^8^ as well as down-regulating the amount of cellular lipids and glucose uptake in adipocytes^9^. It has been demonstrated that glucose contributes a greater proportion of acetyl units to fatty acid biosynthesis in bovine intramuscular adipose tissue^10^. In adipose tissue, ACSL1 is one of the most highly expressed acyl CoA-synthetases involved in the uptake of fatty acids and in triacylglycerol synthesis^11^,^12^,^13^. Selective ACSL1 deficiency in mice caused a 91% loss of muscle ACSL activity^8^ and an 80% loss of adipose ACSL activity^14^. The uptake of^14^C-DHA and^14^C-arichodonic acid (AA) by villous cytotrophoblast cells was found to be highly dependent on the activity of ACSL1, and thus reductions in its expression level were related to decreases in^14^C-DHA and^14^C-AA uptake^15^. The mitochondrial localization of ectopic ACSL1 in epithelial COS cells was found to be sufficient to increase oleate uptake^16^. By contrast, the suppressive effects of EPA on palmitate-induced cytokine production may be mediated at least partly by the suppression of ACSL1 expression^17^. ACSL1 also acts on both unsaturated and saturated fatty acids in intact cells^18^,^19^. Recently, it was shown that ACSL1 helps to control the composition of esterified fatty acid species among the neutral lipids and phospholipids in adipose tissue^14^ as well as the levels of arachidonoyl-CoA and several phospholipids in macrophages^20^,^21^. Although ACSL1 is induced by PPPAα and PPARγ in skeletal muscle and adipose tissue^13^, ACSL1 depletion in adipocytes does not influence the expression of key adipogenic transcription factors such as PPARγ, C/EBPα, and FABP4^9^,^22^.

The expression of ACSL1 is important for regulating the fatty acid composition of bovine skeletal muscle, but the transcription factors that contribute to the control and regulation of its expression have not been characterized. In this study, we first determined the tissue distribution of ACSL1 mRNA and found that bovine ACSL1was highly expressed in subcutaneous adipose tissue and *longissimus thoracis*. Furthermore, we analyzed molecular mechanisms involved in ACSL1 regulation and found that transcriptional activity of ACSL1 gene was dependent on E2F1, Sp1, KLF15, and E2F4 transcription factors. Understanding the transcriptional regulation of ACSL1 may provide further information on regulatory roles of ACSL1 gene in mediating the lipid composition of beef.

## Results

### Detection of ACSL1 expression in bovine tissues and organs

**Figure 1:**
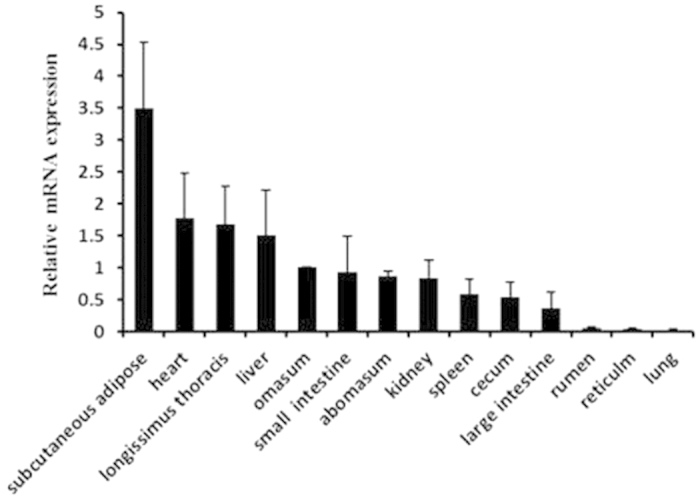
Expression pattern analysis of ACSL1 in bovine tissues and organs. ACSL1 mRNA expression was normalized against that of the housekeeping gene β-actin (ACTB) and expressed relative to gene expression in the omasum. Each column value represents the mean ± standard deviation based on three independent experiments. The error bars denote the standard deviations.

To detect the tissue distribution of bovine ACSL1 mRNA, qPCR was performed using cDNA from 14 bovine tissues and organs:, subcutaneous adipose, heart, *longissimus thoracis*, liver, omasum, small intestine, abomasum, kidney, spleen, cecum, large intestine, rumen, reticulum, and lung tissue. As shown in Fig. 1, the qPCR analysis demonstrated that ACSL1 had a broad tissue distribution among cattle tissues and organs. The basal expression level of ACSL1 was relatively high in subcutaneous adipose tissue and *longissimus thoracis*, but low in the rumen, reticulum, and lung tissue.

### Determination of the transcription start site of ACSL1 gene

**Figure 2:**
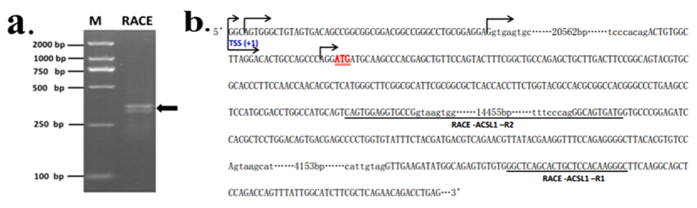
5′ RACE analysis of the ACSL1 cDNA synthesized from skeletal muscle. (**a**) ACSL1 5′ RACE products from nested PCR were analyzed by agarose gel electrophoresis. Arrowheads indicate the resulting DNA bands. (**b**) Sequence of the ACSL1 promoter and the downstream region. The positions of identified TSS were marked with arrows. The primers used for 5′ RACE analysis are underlined. The translational start site (ATG) is shown by a double underline. The 5′ untranslated region and exons are shown in capitals, and introns are shown in small letters with the total intron sizes shown.

In order to analyze the molecular mechanisms involved in ACSL1 regulation, we performed 5′-RACE to identify the TSS of ACSL1 gene. Antisense primer R1 and nested primer R2 (Supplementary Table S1), complementary to the sequence of exon 2 and 3, were used for two successive rounds of PCR. As shown in Fig. 2a, a band of 289 bp was amplified. In total, 15 positive clones had four different 5′ ends in the first and second exons, i.e., at 20659, 20656, 20608, and 3 bp upstream of the translational start site (Fig. 2b). The guanine residue (G) in the proximal 5′ UTR, which was located 20659 bp upstream of the translational start site, was verified as the most upstream the translational start site (TSS-1) and designated as +1. TSS-1 was located 7 bp upstream of the 5′ end of the published ACSL1 mRNA sequence (NM_001076085).

### Isolation of the functional proximal minimal promoter of ACSL1 gene

**Figure 3:**
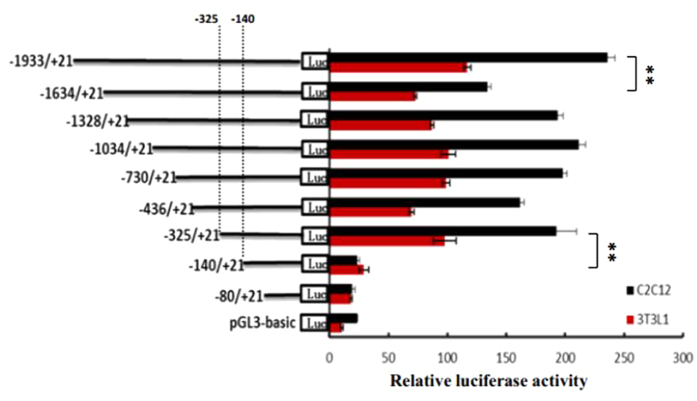
Luciferase activities of the bovine ACSL1 promoter constructs in two cell lines. A series of plasmids containing 5′ unidirectional deletions of the promoter region of the ACSL1 gene (pGL3–1933, 1634, 1328, 1034, 730, 436, 325, 140, pGL3–80, and pGL3) fused in frame to luciferase gene were transfected into 3T3L1 and C2C12 cell lines. After 48 h, the cells were harvested for luciferase assay. Results are expressed as the mean ± standard deviation in arbitrary units based on the firefly luciferase activity normalized against the Renilla luciferase activity for triplicate transfections. The error bars denote the standard deviation. The unpaired Student’s *t*-test was used to detect significant differences. *P < 0.05 and **P < 0.01. The data shown are representative of two independent experiments. Positions −325 and −140 bp in the promoter are also shown.

To measure the activity of potential cis-acting elements and determine the minimum sequence required for activity, a series of nine reporter constructs with progressively larger deletions from the 5′ end of the promoter were generated. The effects of these modifications were evaluated upon transfection of the corresponding luciferase reporter plasmids into 3T3L1 and C2C12 cells, and the results of these analyses are shown in Fig. 3. Luciferase assays revealed a 22-41-fold increased promoter activity of the pGL–1933/+21 as compared to the empty vector in two cell lines, indicating a functional promoter in the −1933/+21 region of ACSL1 gene. When the promoter was deleted to position −1634, the promoter activity with pGL−1634/+12 decreased by ca 37% in 3T3L1 cells and by 43% in C2C12 cells compared with the pGL−1933/+21 (Fig. 3), this result demonstrated that positive regulatory elements were located in the −1933/−1634 region in two cell lines. However, 5′ deletions of the promoter from position −1634 to −325 did not significantly change promoter activity. When the promoter was further deleted to −140 bp, the promoter activity with pGL−325/+12 decreased by ca 75% in 3T3L1 cells and by 93% in C2C12 cells compared with pGL−140/+12. Further 5′ deletions of the promoter from −140 to −80 bp, this plasmid (pGL−80/+12) abolished the promoter activity in two cell lines. These results indicated that the proximal minimal promoter of ACSL1 gene was located within the region −325/−141 relative to TSS-1 (Fig. 3).

### Sequence analysis of the proximal minimal promoter region of ACSL1 gene

**Figure 4:**
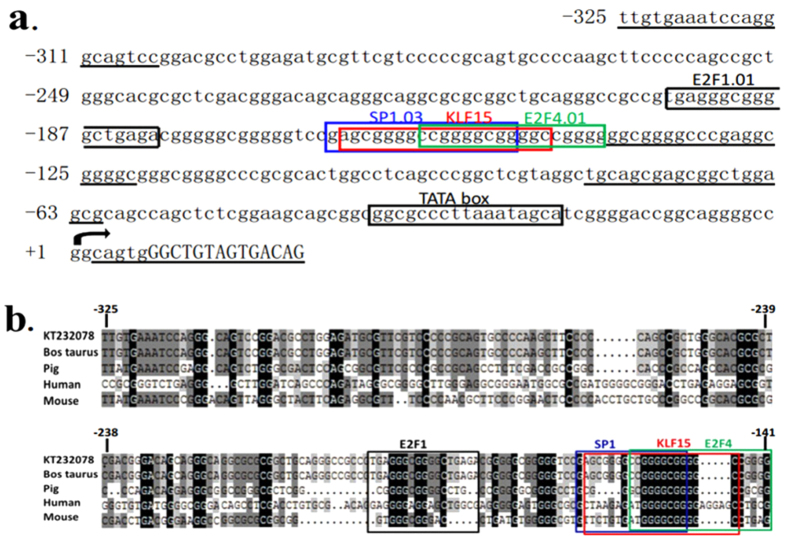
Characterization of the transcription factor-binding sites in the bovine ACSL1 promoter. (**a**) Sequence and putative transcription factor-binding sites of the proximal minimal promoter of ACSL1 gene. ACSL1 transcriptional start site (TSS-1) is indicated by an arrow. The sequence of exon 1 is shown in capitals. Putative transcription factor binding sites are boxed. The primers for unidirectional deletions are underlined. (**b**) Comparison of the sequences in the proximal promoter region of the ACSL1 gene in, cattle, pigs, humans, and mice. The box depicts E2F1, Sp1, KLF15, and E2F4 consensus binding sites.

**Figure 5:**
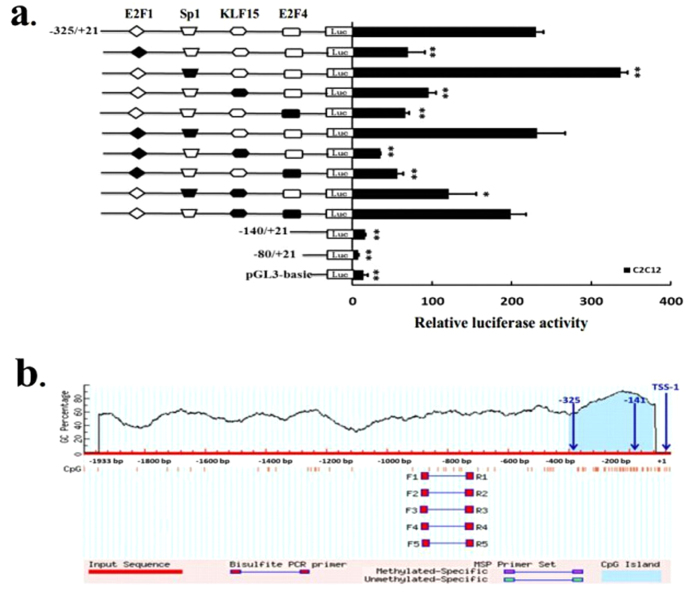
Analysis of E2F1, Sp1, KLF15, and E2F4 binding sites by site-directed mutagenesis and the predicted CpG island in the proximal ACSL1 promoter. (**a**) Site-directed mutagenesis was carried out in the construct pGL−325/+12. The different constructs were transiently transfected into C2C12 cells. After 48 h, the cells were harvested for the luciferase assay. Results are expressed as the mean ± standard deviation in arbitrary units based on the firefly luciferase activity normalized against the Renilla luciferase activity for triplicate transfections. The error bars denote the standard deviation. The paired Student’s *t*-test was used to detect significant differences. *P < 0.05 and **P < 0.01. The data shown are representative of two independent experiments. (**b**) Schematic representation of the proximal promoter region (+1 to −1933 base pairs) of the bovine ACSL1 gene to predict the regions with high GC content. Dashed lines indicate the GC percentage as represented on the y-axis and the x-axis denotes the bp position on the 5′ untranslated region, vertical lines indicate relative positions of CpG dinucleotides. Coordinates are given relative to the translational start site (shown as +1). Arrows indicate the TSS-1, the positions −325 and −141 bp in the promoter.

Analysis to identify regulatory elements in the ACSL1 promoter region was performed using the Matlnspector program ( http://www.genomatrix.com) with a cutoff value over 90%. We identified four consensus motifs as the transcription factor recognition sites for E2F1, Sp1, KLF15, and E2F4 (Fig. 4a,b). In addition, this computer analysis revealed the absence of a CCAAT boxes close to the transcription initiation site (TSS-1). Analysis with MethPrimer program ( http://www.urogene.org/methprimer/) revealed one CpG island located within the region −335/−57 relative to TSS-1, which had a GC content of 78.5% (Fig. 5b).

### E2F1, Sp1, KLF15, and E2F4 are identified as transcriptional activators or repressors in the proximal minimal promoter region of ACSL1 gene

To investigate the roles of these sites in the regulation of ACSL1, we constructed a series of DNA plasmids with 3-bp point mutations in the transcription factor binding sites and transiently transfected them into C2C12 cells. As shown in Fig. 5a, mutation of the Sp1 binding site in the construct pGL−325/+12 resulted in a significant increase in the promoter activity (145%), whereas the mutations of E2F1, KLF15, or E2F4 led to ca 58–71% reductions in the ACSL1 promoter activity. Double mutations of the E2F1 and KLF15 sites resulted in additional reductions in the transcriptional activity compared with the mutations of the E2F1, Sp1, KLF15, or E2F4 sites. These results indicate that the E2F1 site (at position −197 to −181), Sp1 site (at position −165 to −149), KLF15 site (at position −164 to −146), and E2F4 site (at position −157 to −141) were essential for basal transcriptional activity of the ACSL1 proximal minimal promoter. In addition, double mutations of the KLF15 and E2F4 sites resulted in an additional increase in the transcriptional activity compared with the mutations of the KLF15, or E2F4 sites. The reason for this was double mutations of the E2F1 and KLF15 sites created a new binding site for transcription factor (MyT1).

### E2F1, Sp1, KLF15, and E2F4 bind to the proximal minimal promoter of ACSL1 *in vitro*

**Figure 6:**
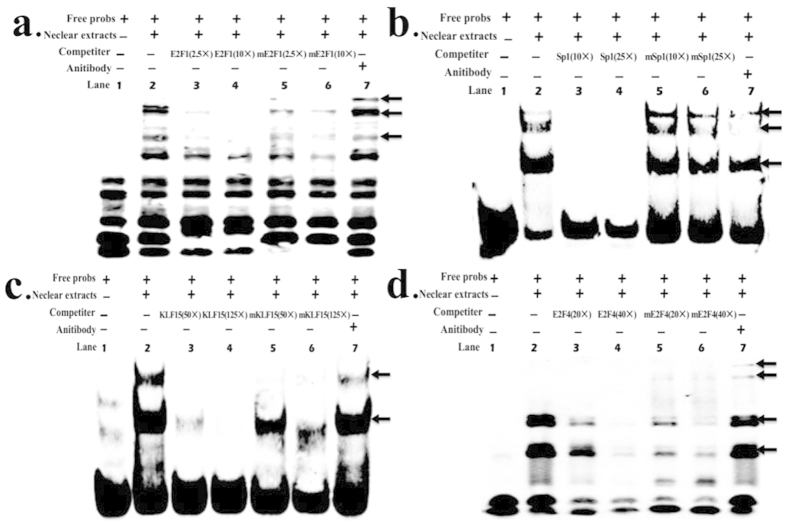
EMSA assays showing direct binding of E2F1, Sp1, KLF15, and E2F4 to ACSL1 promoter *in vitro*. The main complexes are marked with arrows. (**a**) Nuclear protein extracts were incubated with free probe containing the E2F1 binding site in the presence of 2.5× unlabelled probe (lane 3), 10× unlabelled probe (lane 4), 2.5× mutation probe (lane 5), 10× mutation probe (lane 6), or in the absence of any competitor (lane 2). The super-shift assay was conducted using 10 ng anti-E2F1 antibodies (lane 7). (**b**) Nuclear protein extracts were incubated with free probe containing the Sp1 binding site in the presence of 10× unlabelled probe (lane 3), 25× unlabelled probe (lane 4), 10× mutation probe (lane 5), 25× mutation probe (lane 6), or in the absence of any competitor (lane 2). The super-shift assay was conducted using 10 ng anti-Sp1 antibodies (lane 7). (**c**) Nuclear protein extracts were incubated with free probe containing the KLF15 binding site in the presence of 50× unlabelled probe (lane 3), 125× unlabelled probe (lane 4), 50× mutation probe (lane 5), 125× mutation probe (lane 6), or in the absence of any competitor (lane 2).The super-shift assay was conducted using 10 ng anti-KLF15 antibodies (lane 7). (**d**) Nuclear protein extracts were incubated with free probe containing the E2F4 binding site in the presence of 20× unlabelled probe (lane 3), 40× unlabelled probe (lane 4), 20× mutation probe (lane 5), 40× mutation probe (lane 6), or in the absence of any competitor (lane 2). The super-shift assay was conducted using 10 ng anti-E2F4 antibodies (lane 7).

We used electrophoretic mobility shift assays (EMSAs) to confirm whether E2F1, Sp1, KLF15, and/or E2F4 bind to the ACSL1 promoter *in vitro*. As shown in Fig. 6a, the nuclear protein from C2C12 cells bound to the 5′-biotin labeled E2F1 probes and formed three main complexes (lane 2, Fig. 6a). Competition assays verified the specificity of the E2F1/DNA interaction (lanes 3 and 4, Fig. 6a), whereas the mutant probe had little effect on the main complexes (lanes 5 and 6, Fig. 6a). The last lane shows that the complex was super-shifted when it was incubated with E2F1-antibody (lane 7, Fig. 6a). KLF15, Sp1 and E2F4 yielded similar results as E2F1 (Fig. 6b–d). Although these experiments did not reveal a supershifted product at the KLF15 and Sp1 binding sites, the amount of the main complex was clearly decreased (lane 7, Fig. 6b; and lane 7, Fig. 6c).

## Discussion

ACSL1 is located on the mitochondrial outer membrane^8^, a key cellular location for its roles in fatty acid metabolism including fatty acid activation, transport, and degradation, as well as lipid synthesis^6^. In the present study, we first determined the tissue distribution of ACSL1 mRNA and found that bovine ACSL1was highly expressed in subcutaneous adipose tissue and *longissimus thoracis* (Fig. 1), thereby indicating that the ACSL1 gene might play a functional role in mediating the fatty acid composition of bovine skeletal muscle^5^.

To better understand the regulation of ACSL1 at the transcriptional level, we cloned and functionally characterized the promoter of ACSL1 gene. The 5′ flanking sequence of the bovine ACSL1 gene was found to contain four TSSs, a CpG island containing the proximal minimal promoter region, and a consensus TATA box. These observations are not consistent with the fact that the majority of mammalian gene promoters lack a TATA box, although they possess multiple TSSs and CpG islands^23^,^24^. It is possible that the transcription factor binding site of TATA box, which was not located in the proximal minimal promoter region, could be a nonfunctional transcription factor consensus binding site. It is well documented that TATA-driven transcription preinitiation complex assembly is the exception rather than the rule in eukaryotic transcription, as only 10–20% of mammalian promoters contain a functional TATA box^25^. DNA methylation plays an important role in the regulation of gene expression and DNA methylation is essential for normal gene function^26^,^27^. Previously, the ACSL1 gene was shown to be highly associated with histone acetylation during adipocyte differentiation^9^ while histone acetylation and DNA methylation pathways can depend on each other^28^. In addition, E2F1 and E2F4 transcription factors can form complexes with histone deacetylases and histone methyltransferases^29^. In the present study, we found that the recognition sites of E2F1 and E2F4 transcription factors were located in the CpG island containing the proximal minimal promoter of ACSL1 gene. Thus, these results strongly suggest that the ACSL1 gene may be subject to epigenetic regulation.

Analysis of a region comprising positions −1933 to +21 of ACSL1 indicated that the minimal active promoter sequence may be localized to the sequence from −325 to −141, which contains the consensus motifs for the E2F1, Sp1, KLF15, and E2F4 transcription factors (Fig. 4b). E2F transcription factors regulate both proliferative and metabolic genes^30^. E2Fs 1 to 5 all contain both activation and repression domains, yet E2Fs 1, 2, and 3 are frequently classified as E2F activators, whereas E2Fs 4, 5 and 6 are classified as repressors^29^. E2F1 triggers the expression of PPARγ during the early stages of adipogenesis, whereas E2F4 represses the expression of PPARγ in the terminal stage of adipocyte differentiation^31^,^32^. E2F1 also modulates oxidative metabolism in different organs and cell types, and a previous computational analysis of the promoter region of oxidative genes indicated the presence of E2F-binding sites in the regulatory sequences of Cox5a, Cpt-1, Pdk4, Ppargcla, Ucp1, Ucp2, Tfam, Esrra, and Sdha^33^. Furthermore, ACSL1 has a specific function in directing the metabolic partitioning of fatty acids toward β-oxidation in adipocytes^14^. In support of the regulation of oxidative gene transcription by E2F1, we found that mutations of the E2F1 site greatly reduced the activity of the ACSL1 promoter, while EMSA assays also demonstrated that these transcription factors could specifically bind sequences in the proximal promoter of ACSL1 in C2C12 cells. In previous studies, E2F4 has been considered to oppose E2F-1 to -3 as the main repressor in the E2F family, where it mediates cell cycle arrest^34^. However, an increasing number of studies^35^,^36^,^37^,^38^, support the versatile characteristics of this transcription factor, which may switch functions from transcriptional repression to activation or even function as a purely transactivating factor in some settings. Our results strongly support this hypothesis because E2F4 exhibited positive transcriptional effects after mutation according to the EMSA assay. These results demonstrate that E2F1 and E2F4 play important roles in regulating the transcriptional activity of ACSL1 gene, and thus it might contribute to determining the fatty acid composition of bovine skeletal muscle.

Sp1 is known to be act as both a negative and a positive regulator of gene transcription^39^. Sp1 is also known to interact directly with E2F-1 to -3^40^,^41^,^42^, as well as histone deacetylases1 (HDAC1)^40^, but not with E2F4 and E2F5^40^. The cooperation between E2F and Sp1 leads to both repressive and active promoter states, which depend on the recruitment of either HDAC-containing repressor complexes^39^,^43^ or histone acetyltransferases^39^. We observed that mutation of the Sp1 site increased the basal promoter activity and our EMSA results showed that Sp1 was capable of binding with high affinity to this sequence, thereby suggesting that Sp1 could have an inhibitory function. These findings support a previous model^39^ and they suggest that the E2F complex carries p130 most likely bound to E2F4. As E2F4 lacks the sequence required to interact with Sp1, the nearby Sp1 is able to bind with HDAC1. Thus, both Sp1 and p130 recruit HDAC1, thereby leading to the full inactivation of the promoter. In addition, E2F1 may displace HDAC1 from the C terminus of Sp1, but E2F1 itself could interact with retinoblastoma protein (RB), which again recruits HDAC1 or HDAC2 to promoter regions, thus keeping the promoter inactive^39^. However, previous analyses of the c-myc, dihydrofolate reductase (DHFR), and mouse thymidine kinase promoters have shown that the E2F and Sp1 sites interact functionally to cooperatively activate transcription^40^,^41^,^44^, although the mechanism that underlies this role has not been elucidated. Therefore, more detailed studies of the sites of contact and the construction of the transcription factors are required for a full understanding of the biological consequences of interaction between E2F and Sp1.

KLF15 is a member of the Spl-like/KLF family, which are closely related zinc-finger DNA-binding proteins, important regulators of glucose, amino acid metabolism, and lipid utilization^45^,^46^,^47^. KLF15 directly upregulates some glucose transport and metabolic genes, such as glucose transporter 4, acetyl-CoA synthetase 2, and phosphoenolpyruvate carboxykinase^48^,^49^. Bioinformatics/ChIP analyses and loss-of-function experiments strongly suggest that KLF15 directly regulates several important genes in the lipid-flux pathway^47^. KLF15 also plays essential roles in the regulation of skeletal muscle^47^, myocardial lipid flux^50^, and gene transcription during adipogenesis^51^. We found that mutation of the KLF15 site greatly reduced the activity of the promoter, while the EMSA assay indicated that this transcription factor can specifically bind sequences in the proximal promoter of ACSL1 in C2C12 cells. These results demonstrate that KLF15 plays an important role in regulating the transcriptional activity of ACSL1 gene, and thus it might contribute to determining the fatty acid composition of bovine skeletal muscle.

In conclusion, our study revealed that bovine ACSL1 is highly expressed in subcutaneous adipose tissue and *longissimus thoracis* and the expression of the ACSL1 is regulated by multiple transcription factors, including E2F1, Sp1, KLF15 and E2F4. Epigenetic modification in the ACSL1 promoter with an effect on transcription of the gene remains to be investigated. These results may lead to enhanced understanding of the regulation of ACSL1expression in other models, as well as providing new insights into the regulatory mechanisms and biological functions of the ACSL1 gene in mediating the lipid composition of beef.

## Materials and Methods

### Tissue expression profile analysis

Fourteen tissues were obtained from three adult Qinchuan cattle. Total RNA was extracted from the tissues using a Total RNA kit (Tiangen, Beijing, China) and then reverse-transcribed using a PrimeScript™ RT reagent Kit with gDNA Eraser (Perfect Real Time) (TaKaRa, Dalian, China). cDNA from reverse transcription of individual animal samples for each tissue was pooled for qPCR which was performed using a SYBR Green PCR Master Mix kit (TaKaRa, Dalian, China) and 7500 System SDS V 1.4.0 (Applied Biosystems, USA). All of the primers used in the real-time PCR experiment are listed in Supplementary Table S1. The data were normalized against those obtained for the housekeeping gene, β-actin (ACTB), which was used as an endogenous control gene. The relative expression levels of the target mRNAs were calculated using the 2^−ΔΔCt^ method^52^.

### Rapid amplification of cDNA ends (5′ RACE)

The transcription initiation site (TSS) of the bovine ACSL1 gene was determined using a BD SMARTTM RACE cDNA amplification kit (Clontech Inc, CA, USA) according to the manufacturer’s instructions. Briefly, 1 μg of total RNA isolated from the *longissimus thoracis* was reverse-transcribed with PowerScript RT (Clontech Inc, CA, USA). PCR was performed using a Universal Primer A Mix (UPM) (Clontech Inc, CA, USA) and gene-specific primers (Supplementary Table S1) located in exons 2 and 3 of the ACSL1 gene. The primary PCR products were diluted 20-fold as the nested PCR template. The PCR products were separated by electrophoresis on 2% agarose gels containing 0.6 g/mL ethidium bromide and visualized under UV. The purified amplimers were cloned into T-Vector pMD19 (simple) (TaKaRa, Dalian, China) and 20 clones were sequenced.

### Promoter cloning and generation of luciferase reporter constructs

In order to clone the bovine ACSL1 promoter region, we designed gene-specific primers to amplify a 2.0-kb genomic region upstream of the bovine ACSL1 gene TSS. The PCR product of 1954 bp was isolated from agarose gel using a Gel Extraction Kit (omega), and it was cloned into T-Vector pMD19 (simple) and submitted (GenBank No. KT232078). For the generation of the luciferase reporter construct, the 2.0-kb bovine ACSL1 promoter fragment was excised from the T-Vector pMD19 (simple) by digestion with Sac1 and Xho1 (TaKaRa, Dalian, China) and ligated into the pGL3-basic vector digested with the same restriction enzymes. This plasmid was named pGL3-1933. Plasmids pGL3-1328, -1034, -730, -325, -251, -207, -140 and -80, which contained′ unidirectional deletions of the promoter, were generated by PCR using specific primers with the sequence of the Sac1 and Xho1 restriction sites incorporated and pGL3-1933 as template. Substitution mutation constructs were generated by a QuickChange Site-Directed Mutagenesis Kit (Stratagene, La Jolla, CA, USA). The MatInspector programme available online at http://www.genomatix.de software was used to analyze putative transcription factor binding sites on the positive and negative chain of the ACSL1 promoter, and to ensure the site-directed mutagenesis would not create new binding sites of other transcription factors. All the constructs were sequenced in both directions (Jinsirui, Nanjing, China).

### Cell culture and transfection

Two mouse cell lines (C2C12 myoblasts) and 3T3L1 cells were maintained in Dulbecco’s modified Eagle’s medium (DMEM; Invitrogen, USA) with 4500 mg/L glucose, where the medium was supplemented with 10% fetal bovine serum (FBS) (PAA, Austria), 100 units/mL penicillin (final concentration), and 100 mg/mL streptomycin. The cells were incubated at 37 °C with 100% humidity under 5% CO_2_ and passaged using standard cell culture techniques.

Cells were plated at a density of 1.2 × 10^5^ cells/well in 24-well dishes and 24 h later, they were transfected with the plasmids described above using X-tremeGENE HP DNA transfection reagent (Roche, USA) according to the manufacturer’s instructions. Briefly, cells were plated at 80–90% confluence and cultured overnight at 37 °C with DMEM containing 10% FBS, but without antibiotics. The transfection reagent was mixed with opti-MEM (6 μ/150 μL medium) (GIBCO-Invitrogen), incubated for 5 min at room temperature, combined with different plasmids (2.4 μg/150 μL for pGL3 and 0.03 μg/150 μL for pRL-TK), and then incubated for 20 min at room temperature. In total, 100 μL of the DNA-transfection reagent mixture was added to each well (in triplicate). At 48 h after transfection, the cells were washed with PBS and total lysates prepared using passive lysis buffer (Promega Corp). Luciferase activity was measured using the Dual Reporter assay system (Promega Corp) and NanoQuant Plate™ (TECAN, infinite M200PRO). The level of firefly luciferase activity was normalized to Renilla luciferase activity and expressed as arbitrary units.

### Electrophoretic mobility shift assays (EMSA)

Nuclear extracts from C2C12 cells were prepared using the Nuclear Extract Kit (Active Motif Corp., Carlsbad, CA, USA) according to the manufacturer’s protocol. The protein concentration in the nuclear fraction was determined using the Bradford dye assay (Bio-Rad Corp., Richmond, CA, USA). All of the DNA probes used in the EMSA assays (listed in Supplementary Table S1) were synthesized (Invitrogen) and labeled at the 5′ end with biotin. Briefly, 10 μg of nuclear protein extract was incubated with 2 μL 10× binding buffer and 1 μL poly (dI.dC) in a volume of 20 μL for 15 min on ice. Then 200 fmol of the labeled probes were added and the reaction mixture was allowed to incubate at room temperature for 20 min. For the competition assay, unlabeled probes or mutated probes were added to the reaction mixture 15 min before adding the labeled probes. For the super-shift assay, 10 μg each of E2F1, Sp1, KLF15, or E2F4 (Santa Cruz, USA) antibody was added to the reaction mixture and then incubated on ice for 30 min before adding the labeled probes. Finally, the main complexes were resolved by electrophoresis in 6% non-denaturing polyacrylamide gel electrophoresis (PAGE) using 0.5× TBE buffer for 1 h.

## Additional Information

**How to cite this article**: Zhao, Z.-D. *et al*. Characterization of the promoter region of the bovine long-chain acyl-CoA synthetase 1 gene: Roles of E2F1, Sp1, KLF15, and E2F4. *Sci. Rep.* **6**, 19661; doi: 10.1038/srep19661 (2016).

## Supplementary Material

Supplementary Information
