# Supplementary material for: Characterization of the promoter region of the bovine long-chain acyl-CoA synthetase 1 gene: Roles of E2F1, Sp1, KLF15, and E2F4
Source: Sci Rep. 2016 Jan 19;6:19661. doi: 10.1038/srep19661 (PMC4726046; doi:10.1038/srep19661)
Supplement: Supplementary Information [file srep19661-s1.pdf]

Characterization of the promoter region of the bovine acyl-CoA synthetase 1 gene: Roles of E2F1, Sp1, KLF15, and E2F4

Zhi-Dong Zhao[1], Lin-Sen Zan[1, 2, *], An-Ning Li[1], Gong Cheng[1, 2], Shi-Jun Li[1], Ya-Ran Zhang[1], Xiao-Yu Wang[1], Ying-Ying Zhang[1]

1. College of Animal Science and Technology, Northwest A&F University, Yangling 712100 Shaanxi, People's Republic of China.

2. National Beef Cattle Improvement Center, Northwest A&F University, Yangling 712100 Shaanxi, People's Republic of China.

Table S1.
Primers used in the expression and functional analysis of the ACSL1 promoter

Reaction	primer	Primer sequence(5'-3')	Binding region	Size (bp)
qRT-PCR	ACSL1 forward	CTTCGCAGTGGCATCATTAG	+53285/+53305	178
	ACSL1 reverse	GTCCGTAGCCTTCGTAGAAC	+55111/+55131	--
	β-actin forward	CATCGGCAATGAGCGGTTCC	--	147
	β-actin reverse	ACCGTGTTGGCGTAGAGGTC	--	--
Promoter cloning	ACSL1 promoter forward	TGGCAAACTAGTACACCACAGACC	-1933/-1909	1954
	ACSL1 promoter reverse	CTGTCACTACAGCCCACTG	+3/+21	--
	ACSL1 promoter forward 1	***GAGCTC***TGGCAAACTAGTACACCACAGACC	-1933/-1909	1954
	ACSL1 promoter forward 2	***GAGCTC***TTGCTGTACCACGTGCAGGAG	-1634/-1613	1655
	ACSL1 promoter forward 3	***GAGCTC***GGTCGCGAACAGTCAGATATG	-1328/-1307	1349
	ACSL1 promoter forward 4	***GAGCTC***CAGTAGAGGTTATCTGTCAC	-1034/-1014	1055
	ACSL1 promoter forward 5	***GAGCTC***AGAACTTGGCAGGTGGAC	-730/-712	751
	ACSL1 promoter forward 6	***GAGCTC***GCCTGTGGATGAAGGTGGAAG	-436/-415	457
	ACSL1 promoter forward 7	***GAGCTC***TTGTGAAATCCAGGGCAGTCC	-325/-304	346
	ACSL1 promoter forward 8	***GAGCTC***CGAGCTCGGCGGGGCCCGAGGCGGGGC	-140/-120	161
	ACSL1 promoter forward 9	***GAGCTC***CGAGCTCTGCAGCGAGCGGCTGGAGCG	-80/-120	101
	ACSL1 promoter reverse 1	***CTCGAG***CTGTCACTACAGCCCACTG	+3/+21	--
5'RACE	R1	CGGGAACACCTCGTCACGACTCGG	+20843/+35338	507
	R2	GTAGTGACGGGCCGTGGAGGTGAC	+39636/+39660	289
Site-directed mutagenesis	E2F1 forward	GCAGGGCCGCCGTGAGG***AAA***GGGCTGAGACGGGG	-209/-176	346
	E2F1 reverse	CCCCGTCTCAGCCC***TTT***CCTCACGGCGGCCCTGC	--	--
	Sp1 forward	GCGGGGGTCCGAGCG***TTT***CCGGGGCGGGGCCGG	-175/-143	346
	Sp1 reverse	CCGGCCCCGCCCCGG***AAA***CGCTCGGACCCCCGC	--	--
	KLF15 forward	GGTCCGAGCGGGGCC***AAA***GCGGGGCCGGGGGGC	-170/-138	346
	KLF15 reverse	GCCCCCCGGCCCCGC***TTT***GGCCCCGCTCGGACC	--	--
	E2F4 forward	CCGAGCGGGGCCGGGG***TTT***GGCCGGGGGGCGGGG	-167/-134	346
	E2F4 reverse	CCCCGCCCCCCGGCC***AAA***CCCCGGCCCCGCTCGG	--	--
	KLF15-Sp1 forward	GCGGGGGTCCGAGCG***TTT***CC***AAA***GCGGGGCCGG	-175/-143	346
	KLF15-Sp1 reverse	CCGGCCCCGC***TTT***GG***AAA***CGCTCGGACCCCCGC	--	--
	E2F4-Sp1 forward	GCGGGGGTCCGAGCG***TTT***CCGGGG***TTT***GGCCGG	-175/-143	346
	E2F4-Sp1 reverse	CCGGCC***AAA***CCCCGG***AAA***CGCTCGGACCCCCGC	--	--
	KLF15- E2F4 forward	CCGAGCGGGGCC***AAA***G***TTT***GGCCGGGGGGCGGGG	-167/-134	346
	KLF15- E2F4 reverse	CCCCGCCCCCCGGCC***AAA***C***TTT***GGCCCCGCTCGG	--	--
EMSA	E2F1 forward	GCAGGGCCGCCGTGAG***GGCGGG***GCTGAGACGGGG	-209/-176	346
	E2F1 reverse	CCCCGTCTCAGC***CCCGCC***CTCACGGCGGCCCTGC	--	--
	Sp1 forward	GCGGGGGTCCGAGCG***GGGC***C***G***GGGCGGGGCCGG	-175/-143	346
	Sp1 reverse	CCGGCCCCGCCC***C***G***GCCC***CGCTCGGACCCCCGC	--	--
	KLF15 forward	GGTCCGAGCGGGGCC***GGGG***C***G***GGGCCGGGGGGC	-170/-138	346
	KLF15 reverse	GCCCCCCGGCCC***C***G***CCCC***GGCCCCGCTCGGACC	--	--
	E2F4 forward	CCGAGCGGGGCCGGG***GCGG***GGCCGGGGGGCGGGG	-167/-134	346
	E2F4 reverse	CCCCGCCCCCCGGCC***CCGC***CCCGGCCCCGCTCGG	--	--
